# Organic Zinc Salts as Pro-Ecological Activators for Sulfur Vulcanization of Styrene–Butadiene Rubber

**DOI:** 10.3390/polym11101723

**Published:** 2019-10-21

**Authors:** Magdalena Maciejewska, Anna Sowińska, Judyta Kucharska

**Affiliations:** Institute of Polymer and Dye Technology, Lodz University of Technology, Stefanowskiego Street 12/16, 90-924 Lodz, Poland; anna.sowinska@edu.p.lodz.pl (A.S.); 204352@edu.p.lodz.pl (J.K.)

**Keywords:** organic zinc salts, activator, vulcanization, rubber, zinc oxide

## Abstract

Organic zinc salts and complexes were applied as activators for sulfur vulcanization of styrene–butadiene elastomer (SBR) in order to reduce the content of zinc ions in rubber compounds as compared with conventionally used zinc oxide. In this article, the effects of different organic zinc activators on the curing characteristics, crosslink densities, and mechanical properties of SBR as well as the aging resistance and thermal behavior of vulcanizates are discussed. Organic zinc salts seem to be good substitutes for zinc oxide as activators for sulfur vulcanization of SBR rubber, without detrimental effects to the vulcanization time and temperature. Moreover, vulcanizates containing organic zinc salts exhibit higher tensile strength and better damping properties than vulcanizate crosslinked with zinc oxide. The application of organic zinc activators allows the amount of zinc ions in SBR compounds to be reduced by 70–90 wt % compared to vulcanizate with zinc oxide. This is very important for ecological reasons, since zinc oxide is classified as being toxic to aquatic species.

## 1. Introduction

Zinc oxide (ZnO) is widely used as an activator in the vulcanization of unsaturated elastomers by sulfur or sulfur donors. As a result, the amount of bound sulfur and the efficiency of the crosslinking system are enhanced. Moreover, zinc oxide reduces the vulcanization time of rubber compounds and improves the processing and physical properties of vulcanizates [1]. It also used as a crosslinking agent of elastomers containing suitable functional groups, such as carboxylated and chloroprene rubbers [2,3,4] or chlorosulfonated polyethylene [5]. Furthermore, ZnO is used as a filler of rubber composites intended for products exhibiting increased heat conductivity [6]. Despite the important role of ZnO in sulfur vulcanization, its concentration in rubber compounds, especially those used in aquatic environments, must be reduced to, at least, below 2.5 wt %, because zinc oxide is classified as being toxic to aquatic life. According to European Union Regulation (EC) No 1272/2008 on classification, labeling and packaging of substances and mixtures, ZnO was classified as Aquatic Acute 1 with hazard statement H400: Very toxic to aquatic life and Aquatic Chronic 1 with hazard statement H410: Very toxic to aquatic life with long lasting effects. The precaution recommended in this Regulation is defined as P273: Avoid release to the environment. The release of zinc from rubber products occurs during their manufacture, use (dust created during the abrasion of tires on road surfaces) and the recycling or disposal in landfills. A potential source of zinc in groundwater can also be rubber granulates made from end-of-life tires used to build artificial sports fields. Taking this into account, the problem of reducing the amount of zinc in rubber products becomes essential. Methods to reduce the amount of ZnO in elastomers have been widely studied. Chapman and Johnson [7] replaced traditionally used microsized ZnO with more active forms of zinc oxide characterized by a high surface area or very small particle size. This allowed for the reduction of ZnO content to 2 parts per hundred of rubber (phr) in ethylene–propylene–diene monomer rubber (EPDM) or natural rubber (NR) compounds. Further reduction of the zinc oxide level led to reversion during vulcanization and deterioration of mechanical properties such as the modulus or compression set of vulcanizates.

Pysklo et al. [8] reported that the amount of ZnO can be reduced in carbon black-filled polyisoprene rubber (IR) to 2 phr and in styrene–butadiene elastomer (SBR) compounds to 1 phr without affecting the vulcanization kinetics and mechanical properties of the vulcanizates. The crown ether 18-crown-6 was used as the interface transfer catalyst to increase the rate and efficiency of the crosslinking reaction. Lower than 1–2 phr content of ZnO significantly increased the reversion during vulcanization.

Maciejewska and Zaborski [9] used zinc oxide nanoparticles to reduce the content of ZnO in acrylonitrile–butadiene elastomer (NBR) filled with nanosized silica. Replacing 5 phr of microsized ZnO with 2 phr of zinc oxide nanopowder did not affect the curing behavior of NBR and consequently the crosslink density or tensile properties of vulcanizates. Moreover, vulcanizates with nanosized ZnO demonstrate better damping properties and higher thermal stability compared with the vulcanizate that contains a standard activator.

Kim et al. incorporated nanosized ZnO instead of conventional ZnO into NR/butadiene rubber (BR) compounds filled with silica [10]. NR/BR composites containing 0.3–3 phr of nanosized ZnO showed improved cure characteristics, lower optimum vulcanization time, higher tensile strength and tear strength compared to the composite crosslinked with 5 phr of microsized ZnO.

According to the generally accepted mechanism of sulfur vulcanization, zinc cations from ZnO react with accelerators, forming active zinc-accelerator complexes, which are believed to be more reactive than free accelerators [1,11,12]. In order to increase the activity of ZnO at this step of vulcanization, the availability of zinc ions for reaction with the accelerator should be increased. Owing to the crystalline structure of ZnO, zinc ions inside the crystals have limited accessibility. Therefore, to reduce the amount of zinc ions in rubber compounds, it is necessary to apply more chemically active zinc, in the form of reactive complexes, in which the availability of zinc ions is higher than in the crystals of ZnO.

Heideman et al. used zinc stearate, zinc 2-ethylhexanoate, and zinc m-glycerolate to activate the sulfur vulcanization of EPDM and styrene–butadiene elastomer (SBR) [13]. The highest vulcanization activity is exhibited by zinc m-glycerolate which has the highest amount of zinc ions per 1 g of activator. It seems to be a good substitute for ZnO without detrimental effects on the crosslinking degree of elastomers, their mechanical properties, and resistance to thermo-oxidative aging. However, it should be noted that in the case of rubber compounds crosslinked with zinc m-glycerolate, the content of zinc ions is higher than in the rubber compound containing ZnO, so no reduction in the zinc amount is achieved. Other zinc complexes show lower efficiency as activators than ZnO, probably due to the considerably lower content of zinc ions introduced into rubber corresponding to ZnO. They result in a much lower crosslinking degree of elastomers compared with the compound containing zinc oxide.

Another approach to reduce the amount of zinc oxide in rubber compounds is the development of zinc loaded clay (Zn-clay) as a vulcanization activator with improved zinc ion accessibility [14]. This activator consists of montmorillonite clay into which zinc ions are introduced via the ion exchange process. Zn-clay achieves comparable SBR curing characteristics, crosslinking degree, and mechanical properties to the conventional system with zinc oxide. The content of zinc ions in the SBR compounds is reduced by a factor of 10–20 compared with the ZnO activator.

Przybyszewska et al. developed new zinc chelates with 1,3-diketones as activators for the sulfur vulcanization of NBR. These chelates are synthesized using commercially available diketones, according to a simple procedure [15]. The application of zinc chelates in the crosslinking process provides vulcanizates with crosslink densities similar to those with 2 phr of nanosized ZnO. This reduced the amount of zinc ions applies by more than 70 wt % compared with nanosized zinc oxide. Moreover, the most active zinc chelates, such as zinc 1,3-diphenylpropane-1,3-dione and zinc 1-(4′-t-butylphenyl)-3-phenylpropane-1,3-dione, considerably reduce the optimal time of NBR vulcanization and allow vulcanizates with significantly higher tensile strength and elasticity to be obtained compared to using nanosized ZnO. However, despite the high activity of zinc chelates with 1,3-diketones, the necessity of synthesis is a limitation on their use on a larger scale.

In this work, we applied commercially available organic zinc salts and complexes, such as zinc gluconate (GluZn), zinc ricinoleate (RicZn), and zinc acetylacetonate (AAcZn) to activate the sulfur crosslinking of SBR rubber. The goal of this research was to develop pro-ecological substitutes for zinc oxide that allow for a reduction in the amount of zinc ions in the rubber compounds.

Pursuant to the (EC) No 1272/2008 Regulation, AAcZn is not considered as a substance harmful to the environment. It is widely used in polymer technology as an initiator for lactide polymerization [16] and for polymerization of 1,3-trimethylene carbonate or 2,2-dimethyltrimethylene carbonate resulting in the production of polycarbonates for medical applications (e.g., medical implants) [17]. AAcZn is also used as a curing catalyst and modifier of epoxy resins [18]. On the other hand, GluZn has been classified by U.S. Food and Drug Administration (FDA) as a generally recognized as safe (GRAS) substance—Subpart I—Nutrients [19]. It is commonly used as a one of the most effective zinc supplements to treat and to prevent zinc deficiency [20]. GluZn is thought to be an antiviral agent or cosmetic biocide effective for use in cosmetics and personal care products. Besides, GluZn can be administrated in pharmaceutical doses in adjunctive therapy of inflammatory acne [21], immune disturbances and common cold [22], dysgeusia [23] or olfactory disorders [24]. Regarding RicZn, it has a very good ability for the adsorption of odor-active compounds, so it is widely used as an odor-removing agent in cosmetic and hygienic applications, such as in surfactant and detergent mixtures, deodorants, perfumes, and toilet preparations [25,26]. Kuhn et al. [27] reported that RicZn exhibits excellent selectivity for organic sulfur and nitrogen compounds. It is able to bind these molecules chemically resulting in the removal of unpleasant odor-active substances from the ambient air. This property of RicZn is also important for its use as a vulcanization activator because most vulcanization accelerators are sulfur and nitrogen compounds. Owing to cosmetic and hygienics applications, the toxicological and dermatological properties of RicZn have been studied. The Cosmetic Ingredient Review (CIR) Expert Panel has classified RicZn as safe and has recommend its use as an anticaking agent, deodorant, and opacifying agent [28].

Therefore, we proposed to use the organic zinc complexes and salts as a pro-ecological alternative for zinc oxide. These compounds were classified as safe and are commonly used even in products approved for contact with human body. Moreover, these organic zinc salts contain less amount of zinc in 1 g of the compound, so the amount of zinc released into the environment from rubber products should decrease significantly. It is believed that organic zinc activators can be dispersed more easily in the elastomer matrix than microsized ZnO, and therefore, the accessibility of zinc ions for reaction with the accelerator is higher, although applications of such organic zinc compounds as activators of SBR vulcanization have not yet been reported. 

## 2. Materials and Methods

### 2.1. Materials

**Scheme 1:**
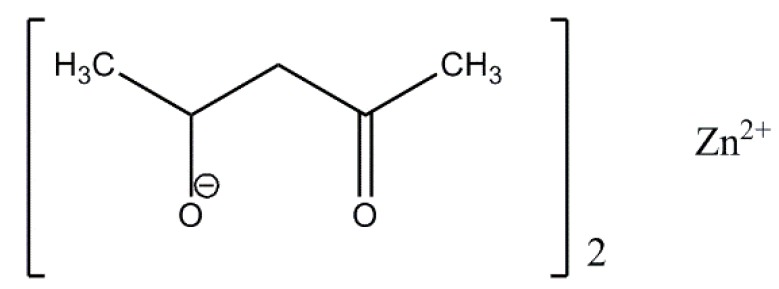
Structure of zinc acetylacetonate (AAcZn).

**Scheme 2:**
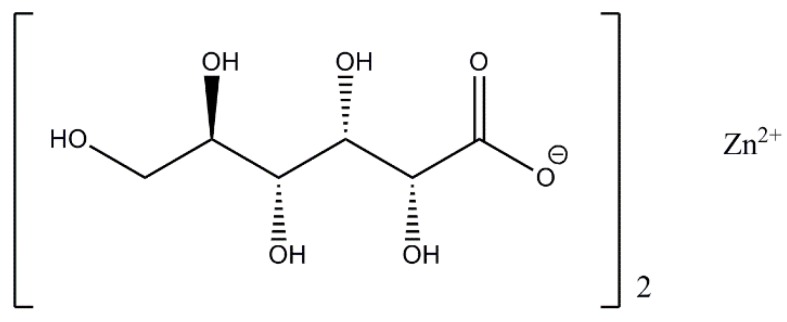
Structure of zinc gluconate (GluZn).

**Scheme 3:**
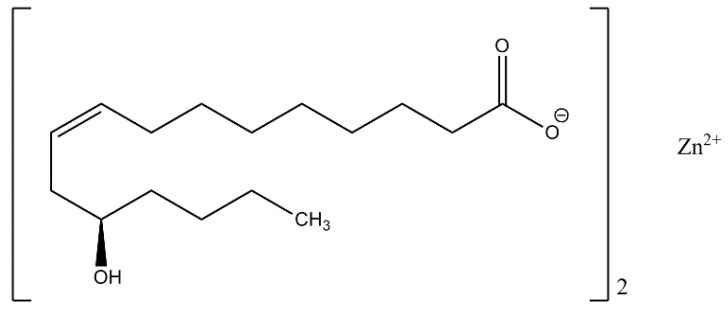
Structure of zinc ricinoleate (RicZn).

SBR (KER 1500) rubber containing 23.5% bonded styrene was obtained from Synthos SA (Oswiecim, Poland). Its Mooney viscosity was ML1+4 (100 °C): 50. SBR rubber was cured using a conventional curing system containing sulfur (Siarkopol, Tarnobrzeg, Poland) in the presence of 2-mercaptobenzothiazole (MBT) and N-cyclohexyl-2-benzothiazolesulfenamide (CBS) as accelerators (Sigma-Aldrich, Schelldorf, Germany). Microsized zinc oxide (ZnO) with a specific surface area of 10 m^2^·g^−1^ (Huta Bedzin, Bedzin, Poland) was applied as activator only for the reference rubber compound. Organic zinc salts and complexes, i.e., GluZn with melting point *M*_p_ 175 °C (Merck, Darmstadt, Germany), RicZn with *M*_p_ 89 °C (Evonik Industries, Essen, Germany) and AAcZn with *M*_p_ 177 °C (Riedel-de Haën, Seelze, Germany) were used as alternative vulcanization activators to ZnO. Carbon black N550 provided by Brenntag Polska (Kedzierzyn-Kozle, Poland) was used as a filler. The structures of applied organic zinc activators are presented in Scheme 1, Scheme 2 and Scheme 3.

### 2.2. Preparation and Characterization of SBR Compounds

Using a two-step procedure, SBR compounds with the compositions given in Table 1 were prepared with a laboratory two-roll mill. First, a masterbatch was prepared containing the sulfur, the vulcanization accelerators CBS and MBT, and the filler. Next, the masterbatch was divided into seven equal pieces, and organic zinc activators were added to each of these pieces, with the exception of the reference rubber compound, where ZnO was introduced. SBR rubber compounds were cured at 160 °C using the optimal vulcanization time (t_95_), which was determined according to the standard PN-ISO 3417:1994 with the D-RPA 3000 rheometer (MonTech, Buchen, Germany).

A differential scanning calorimetry (DSC1) analyzer (Mettler Toledo, Greifensee, Switzerland) was employed to determine the range of SBR vulcanization temperatures and the enthalpy of this process. Measurements were performed in the temperature range of –100 to 250 °C, with a heating rate of 10 °C/min. Nitrogen was used as the protective gas at a flow rate of 80 mL/min. Prior to the measurements, the DSC analyzer was calibrated using indium and n-octane as standards. Liquid nitrogen was applied as cooling agent.

According to the standard PN-74/C-04236, the crosslink density of SBR vulcanizates was determined by their equilibrium swelling in toluene, based on the Flory–Rehner equation [29]. The crosslink density was calculated using the Huggins parameter of elastomer–solvent (toluene) interaction [30]

*µ* = 0.37 + 0.56*V_r_* (1)

where *V_r_* is the volume fraction of elastomer in swollen gel.

The tensile properties of SBR vulcanizates were measured according to the ISO-37 standard procedures using a ZWICK Roell 1435 (Zwick, Ulm, Germany) universal machine. Dumb-bell specimens with a test length of 20 mm and a width of 4 mm were used for the measurements. Uniaxial tensile cyclic loading–unloading experiments were performed at room temperature using the same instrument and samples with the same characteristics. The specimens were stretched to 200% strain and then unloaded to a stress-free state. The loading–unloading cycle was repeated five times and residual strain (permanent set) was produced. 

The hardness of disc-shaped SBR materials was determined by Shore’s method, according to the standard PN-ISO 868, with an indenter that complies with the standard PN-93/C-04206.

The thermo-oxidative aging of the vulcanizates was performed at 100 °C for seven days, according to PN-82/c-04216. To estimate the resistance of the materials to aging, their mechanical properties and crosslink densities were determined after aging and compared with the values obtained for the vulcanizates before the aging process. The aging factor AF was calculated as the change in the deformation energy of the samples according to Equation (2) [31], where TS is the tensile strength of the vulcanizates and EB is the elongation at break:

(2) $$AF=\frac{{\left(EB\cdot TS\right)}_{after\;aging}}{{\left(EB\cdot TS\right)}_{before\;aging}}$$

Thermogravimetry (TG) was employed to study the thermal stability of organic zinc activators and vulcanizates. Measurements were performed using a TG/DSC1 analyzer (Mettler Toledo, Greifensee, Switzerland), previously calibrated with standards (indium, zinc). Measurements were carried out in an argon atmosphere (flow rate 50 mL/min), in the temperature range of 25–600 °C, with a heating rate of 20 °C/min. Next, the gas was changed into air (flow rate 50 mL/min) and samples were heated to 900 °C with the same heating rate. 

Dynamic mechanical measurements were performed in tension mode using a DMA/SDTA861e (Mettler Toledo, Greifensee, Switzerland) analyzer. Measurements were carried out in the temperature range of –100 to 80 °C with a heating rate of 3 °C/min, a frequency of 5 Hz, and a strain amplitude of 10 µm. The temperature of the elastomer glass transition (*T*_g_) was indicated from the maximum of the tan δ = f(T) curve, where tan δ is the loss factor, and T is the measurement temperature.

SEM images of SBR vulcanizates were taken using an LEO1450 scanning electron microscope (Carl Zeiss AG, Oberkochen, Germany). Prior to the measurement, vulcanizates were broken down using liquid nitrogen. Next, their fractures were coated with a carbon layer and tested. Based on the SEM images, the dispersion of activators and filler particles in the SBR elastomer matrix was analyzed. 

## 3. Results and Discussion

### 3.1. Cure Characteristics of SBR Compounds and Crosslink Densities of Vulcanizates 

Rheometer measurements were performed to study the activity of organic zinc salts and complexes during the sulfur vulcanization of SBR compounds. Based on the results, the influences of organic zinc activators on the torque increment during vulcanization, as well as the optimal vulcanization time and scorch time of SBR were determined. Additionally, the crosslink densities of vulcanizates, which correspond to the torque increment during vulcanization of rubber compounds, were determined. The cure characteristics of SBR compounds and crosslink densities of vulcanizates are given in Table 2.

From the presented data, it follows that SBR compounds crosslinked in the presence of organic zinc activators exhibited lower torque increment during vulcanization compared to that of the SBR containing conventional microsized ZnO. The torque increment is an indirect measure of the degree of elastomer crosslinking. Lower values of ΔS during vulcanization fully correlate with smaller crosslink densities of vulcanizates containing organic zinc activators. The types and amounts of organic zinc activator did not affect the torque increment considerably and, consequently, the crosslink density of vulcanizates. The lower activity of organic activators in the crosslinking process, resulting in a lower crosslink density of vulcanizates, was due to a much smaller amount of zinc ions introduced into the rubber in the form of organic zinc salts or complexes compared to ZnO. The amount of zinc ions introduced into rubber in the form of particular activators was calculated using their molecular masses and is shown in Table 2. Depending on the type and amount of used organic zinc activator, SBR compounds contained 70–90 wt % less zinc ions in comparison with the conventionally used 5 phr of ZnO. However, such a small amount of zinc ions seemed to be sufficient to activate sulfur vulcanization of SBR. It should be noticed, that despite the much lower content of zinc ions, organic zinc activators had no detrimental effect on the scorch time or optimal vulcanization time of SBR compounds compared to the conventional ZnO system. This is very important for the safety of rubber compound processing and economical aspects of vulcanization. The optimal vulcanization time of SBR crosslinked with ZnO was 3 min., whereas for organic zinc activators, t_95_ was in the range of 2.5–4.0 min.

**Figure 1:**
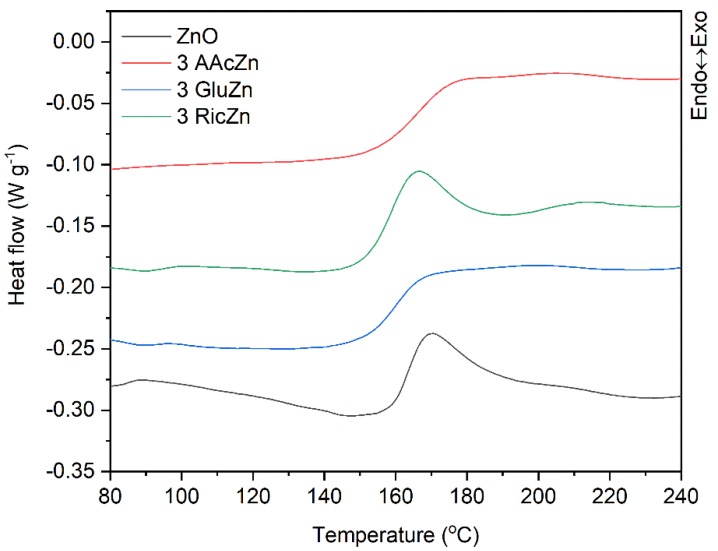
Differential Scanning Calorimetry (DSC) curves of SBR compounds with 3 phr of varying activators.

**Figure 2:**
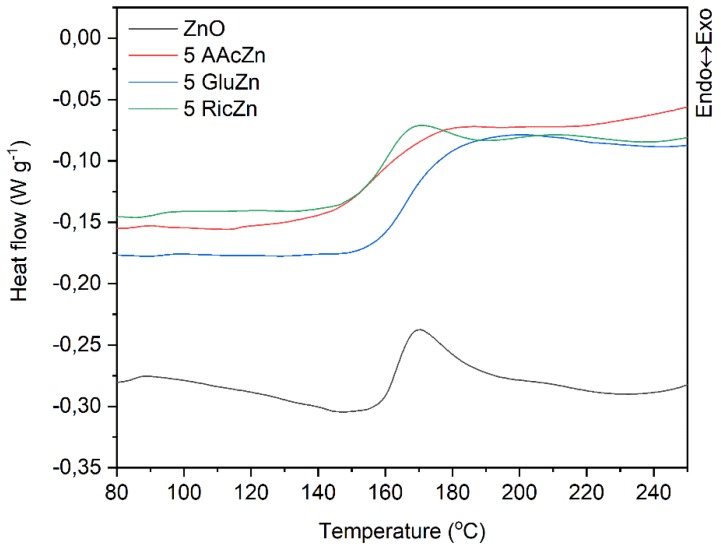
DSC curves of SBR compounds with 5 phr of varying activators.

Having established the influences of organic zinc salts and complexes on the curing characteristics of SBR compounds, we then studied their influences on the temperature and enthalpy of vulcanization using DSC analysis. The results for SBR compounds are presented in Table 3 and Figure 1 and Figure 2.

The vulcanization of the reference SBR compound containing ZnO is an exothermic process that occurs in a wide temperature range of 159–218 °C with a peak temperature of 171 °C and an enthalpy of 10.6 J/g. RicZn did not significantly affect the range of vulcanization temperature compared to that of the rubber compound with conventional microsized ZnO. However, widening of the vulcanization temperature range was observed for SBR cured with AAcZn or GluZn, for which the peaks of vulcanization in DSC curves were broad, fuzzy, and asymmetrical. The endset vulcanization temperature for SBR crosslinked with ZnO was 218 °C, whereas a higher endset temperature was determined for the vulcanization of the rubber compound containing AAcZn (229 °C) or GluZn (232 °C). Using 5 phr of these activators increased also the temperature of the vulcanization peak by 13 °C. So, regarding these parameters, 3 phr of studied organic zinc activators was enough to activate the sulfur vulcanization of SBR. Organic zinc salts and complexes had no detrimental effect on the enthalpy of vulcanization. The ΔH values for SBR crosslinked with ZnO or organic zinc activators were equivalent within the range of experimental error.

Considering the structure of the organic zinc activators, AAcZn is a zinc chelate complex in which acetylacetonate anion complexes by bonding each oxygen atom to the Zn^2+^ cation and forms a chelate ring, which may decrease the availability of zinc for reaction with vulcanization accelerators [32]. On the other hand, GluZn is a zinc salt of gluconic acid comprised of two gluconic acid molecules for each Zn^2+^ cation, whereas RicZn is a zinc salt of ricinoleic acid, which is unsaturated fatty acid. Although the organic zinc activators have different functional groups and molecular mass and, consequently, different content of zinc in 1 g of the compound, their use resulted in a similar time, temperature, and enthalpy of vulcanization, and the crosslink density of SBR. The differences between the results obtained for organic activators were within the error of measurements. However, taking into account the content of zinc ions, it was concluded that RicZn exhibited the greatest activity during vulcanization. Despite the smallest amount of zinc, RicZn allowed the cure characteristics and crosslink density similar to the elastomer crosslinked with AAcZn or GluZn as activators to be obtained. The ability for reaction with organosulfur and nitrogen compounds, which was reported by Kuhn et al. [27], can also contribute to the best activity of RicZn, since vulcanization accelerators contain sulfur and nitrogen atoms in their molecules. Moreover, the melting point of RicZn is 89 °C, so opposite to AAcZn or GluZn it is in a molten state during vulcanization of SBR compounds at 160 °C. As a result, its mobility in the elastomer matrix during vulcanization was higher than AAcZn or GluZn, which are solids at vulcanization temperature. This facilitates contact between the activator and the particles of sulfur or accelerators, which is crucial to activate the vulcanization.

### 3.2. Tensile Properties and Hardness of SBR Vulcanizates

**Figure 3:**
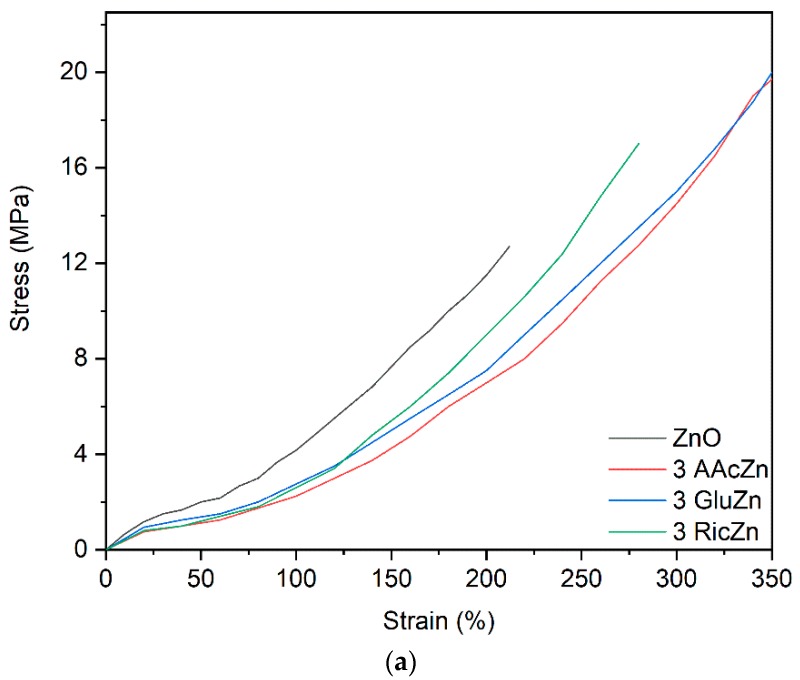

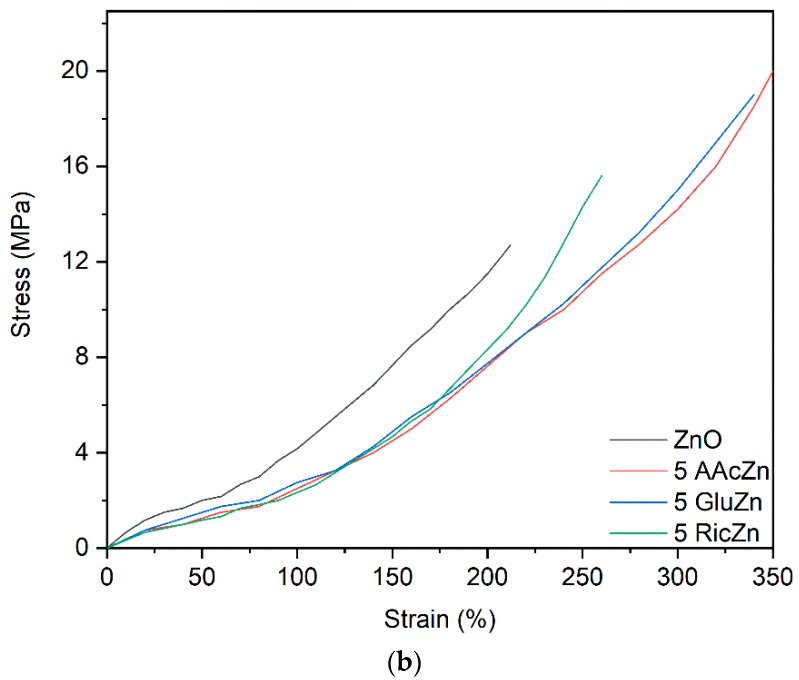
Stress–strain curves of SBR vulcanizates with varying activators: (**a**) 3 phr, (**b**) 5 phr.

It is commonly known that the crosslink density affects the mechanical properties of vulcanizates. By analyzing the values of torque increment during vulcanization and the crosslink densities of SBR vulcanizates, it was concluded that organic zinc activators reduced the amount of crosslinks in the elastomer network compared to the conventionally used 5 phr of microsized ZnO, which can affect the tensile properties and hardness of the vulcanizates. Therefore, the influences of organic zinc salts and complexes on mechanical properties, such as the stress at 100% relative elongation, tensile strength, elongation at break, and hardness of vulcanizates were also examined. The results for SBR vulcanizates are presented in Table 4, whereas the stress–strain curves are shown in Figure 3.

From the data compiled in Table 4, it follows that vulcanizates with organic zinc salts or complexes exhibited approximately 1.2–1.8 MPa lower modulus at relative elongation of 100% (SE_100_) and about 6–11 ShA lower hardness compared to the reference vulcanizate containing ZnO. This was due to their lower crosslink densities. However, these vulcanizates can be still classified as a medium hardness rubber. 

On the other hand, organic zinc activators had no detrimental effect on the tensile strength or elasticity of the obtained vulcanizates. The SBR elastomer crosslinked with conventionally used microsized ZnO exhibited a tensile strength of 12.3 MPa and an elongation at break of 212%. Vulcanizates containing organic zinc activators demonstrated much higher tensile strength, which should be attributed to their low crosslink density. The crosslink density is known to have a significant influence on the mechanical properties of vulcanizates. The decrease in the crosslinks density results in the increase in the elongation at break, and consequently because of the low elongation at break, the TS of vulcanizates increases with the elongation at break. As far as the TS of vulcanizates was concerned, the most effective appeared to be AAcZn and GluZn, for which TS values of approximately 20 MPa and 19 MPa were obtained. On the other hand, the tensile strength of vulcanizate containing RicZn was about 2–3 MPa lower than for other organic zinc activators. The content of organic activator had no influence on the tensile strength of the vulcanizates. It is worth noticing that vulcanizates crosslinked with organic zinc activators, especially AAcZn and GluZn, had a significantly higher elongation at break corresponding to their higher elasticity. The EB values of these vulcanizates were in the range of 338%–350%, opposite to RicZn-containing vulcanizates, for which the elongation at break was 240%–280% due to higher crosslink density compared to vulcanizates with AAcZn or GluZn. 

**Figure 4:**
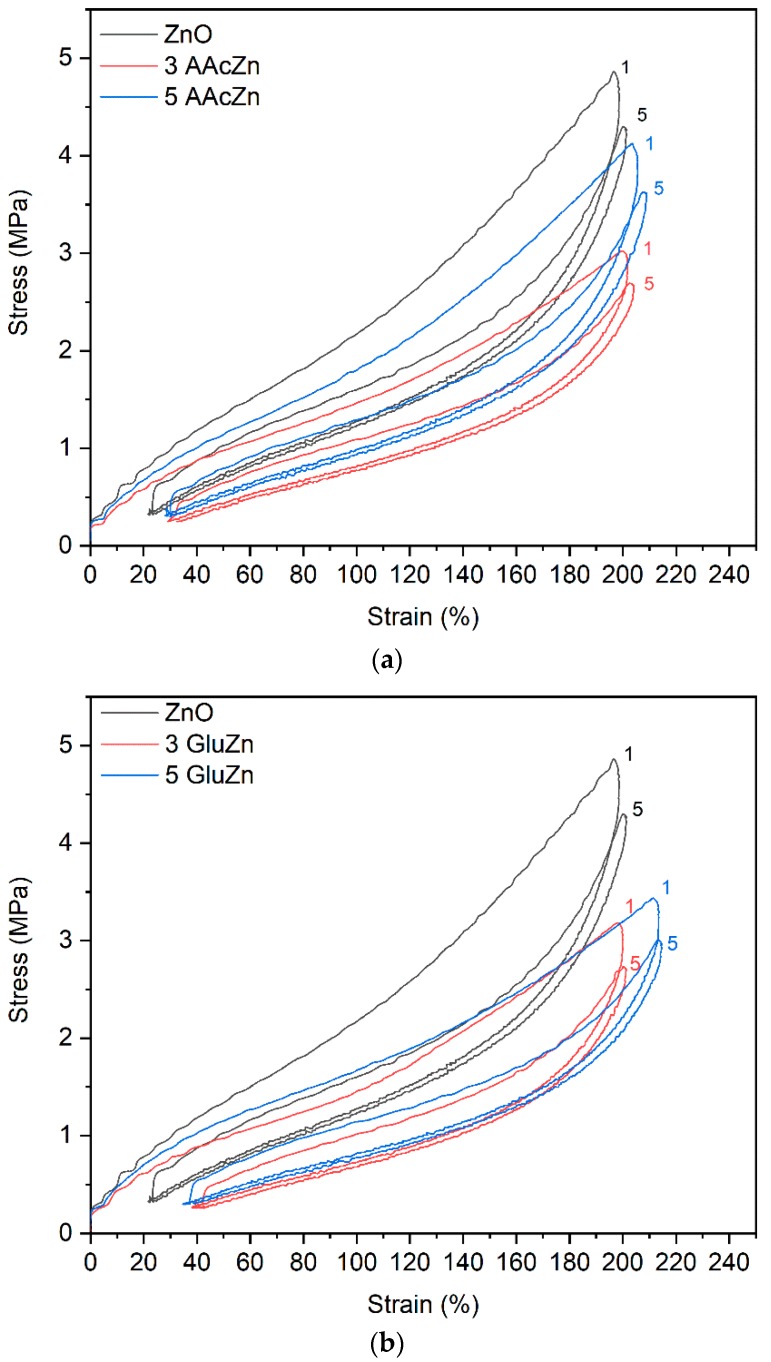

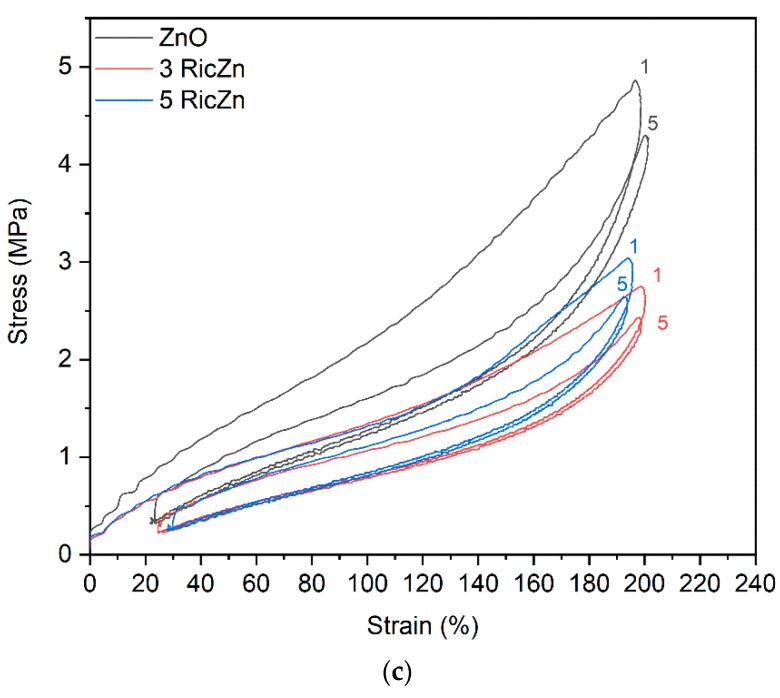
Stress–strain cyclic loading–unloading curves of SBR vulcanizates with varying activators: 1, 5—first and fifth loading–unloading cycle, respectively: (**a**) AAcZn, (**b**) GluZn, (**c**) RicZn.

Cyclic loading–unloading experiments were performed to study the effect of organic zinc activators on the permanent set of SBR vulcanizates. The obtained stress–strain curves for the first and fifth loading–unloading cycles are presented in Figure 4.

For the loading–unloading cycle, the sample was first loaded to a 200% strain and then unloaded, and the cycle was repeated five times. Considering the strain values, the unloading curve in each cycle was significantly lower than the corresponding loading curve. Furthermore, the loading curve in each subsequent cycle recovered with a bigger strain. The sample did not fully return to its original state when unloaded to a zero stress. The residual strain (permanent set) was produced and determined as the difference between the strain of the last unloading curve and the first (virgin) loading curve [33]. The results for SBR vulcanizates are presented in Table 5.

The residual strain after cyclic loading–unloading experiment of the SBR elastomer crosslinked with microsized ZnO was 22%. Owing to lower crosslink densities of vulcanizates, the permanent set was shown to be 5%–15% grater when organic zinc activators were used. The lowest permanent set demonstrated vulcanizates with AAcZn, whereas the highest was determined for the GluZn-containing elastomer. The content of organic zinc activators did not affect the permanent set of the vulcanizates. The residual strain of all tested vulcanizates disappeared during storage of the samples for 30 minutes after the end of the loading–unloading experiment.

The studied vulcanizates are filled with carbon black. Therefore, analyzing the strain–stress curves of cyclic loading–unloading presented in Figure 4, the stress-softening phenomenon and stress hysteresis were observed, which were particularly evident in filled vulcanizates [34]. The unloading and reloading curves of studied vulcanizates did not follow the same path and were situated far from the virgin curve, with the subsequent relaxation paths being located each below the previous one. The subsequent load needed to produce the same deformation of the vulcanizate was smaller than that required during the first loading. The stress-softening phenomenon has been extensively studied [35,36]. According to Dannenberg [37] during primary loading of a vulcanized elastomer, which is composed of macromolecular chains, some of the molecular crosslinks or bonds between chains (entanglements) undergo slippage and breaking. During subsequent stress relaxation, some of the broken bonds rearrange and the slippage partially recovers to its original position. Stress-softening is also interpreted as being caused by sliding between polymeric chains and fillers. The macromolecular chains connected to the reinforcing filler particles are slipping and sliding with friction on these particles [35]. As a result, under cyclic loading conditions, studied vulcanizates demonstrated a loss of stiffness resulting in the stress-softening and stress hysteresis. Therefore, the change in the hard and soft domain microstructure can be expected in the studied material, leading to the increase in the soft domain fraction. Considering organic zinc activators, vulcanizates with AAcZn during the cyclic loading–unloading test showed the most similar behavior to the reference sample with ZnO. On the other hand, vulcanizates with RicZn, which exhibited higher crosslink density than the AAcZn or GluZn-containing elastomer, demonstrated the narrowest stress hysteresis and the smallest differences between the stress required to produce the strain of 200% in the first and in the fifth loading–unloading cycle. Therefore, this vulcanizate exhibited smaller stress-softening under cyclic loading conditions than the rest of the samples tested, resulting from smaller changes in the structure of the elastomer network formed in the vulcanization process, or the internal network formed by filler particles interacting with each other and with elastomer chains.

In summary, despite lower crosslink densities, vulcanizates containing organic zinc activators showed higher tensile strength and elasticity compared to the reference SBR vulcanizate with microsized ZnO. Considering the effects on the mechanical properties and hardness of vulcanizates, the use of 5 phr of organic zinc activators was unreasonable, because it did not improve these properties. Therefore, the amount of organic zinc activators could be reduced to 3 phr, thus reducing the amount of zinc ions in rubber compounds by 80–90 wt % compared to 5 phr of microsized zinc oxide. However, it should be noted that vulcanizates obtained with organic zinc activators were softer and less stiff, so they showed a slightly different behavior than the material cured with ZnO. Therefore, these vulcanizates cannot be regarded as a replacement for ZnO-containing material in certain applications.

### 3.3. Mechanical Properties of SBR Vulcanizates under Dynamic Conditions

**Figure 5:**
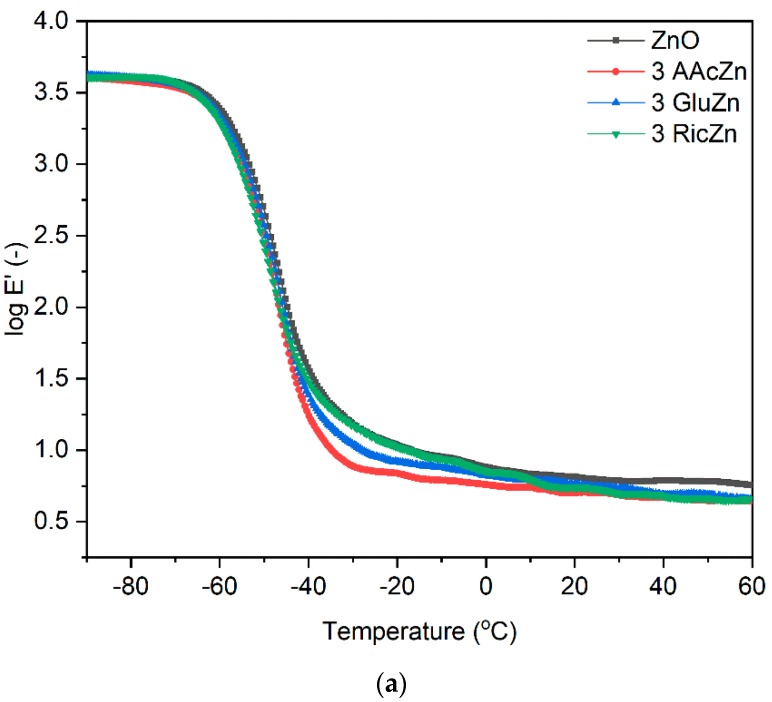

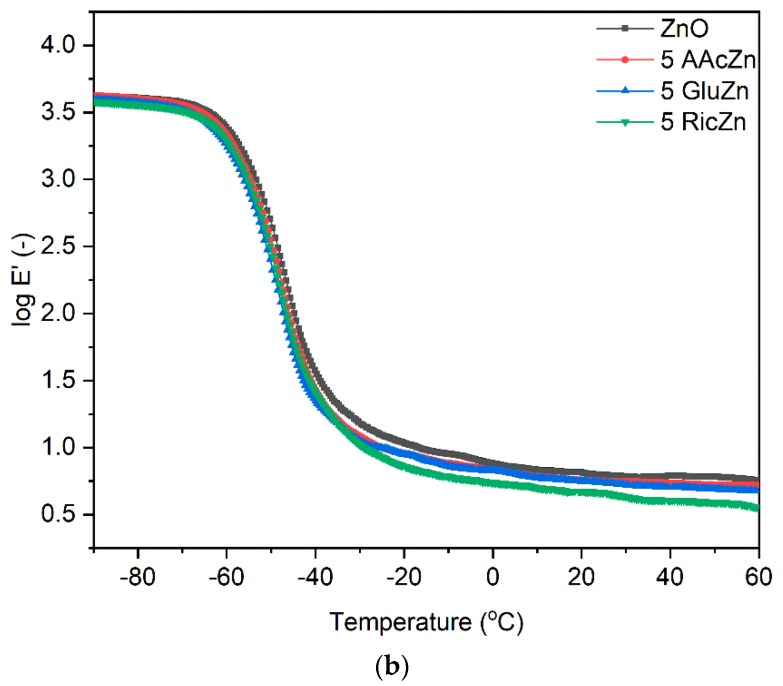
Storage modulus versus temperature of SBR vulcanizates with varying activators: (**a**) 3 phr, (**b**) 5 phr.

**Figure 6:**
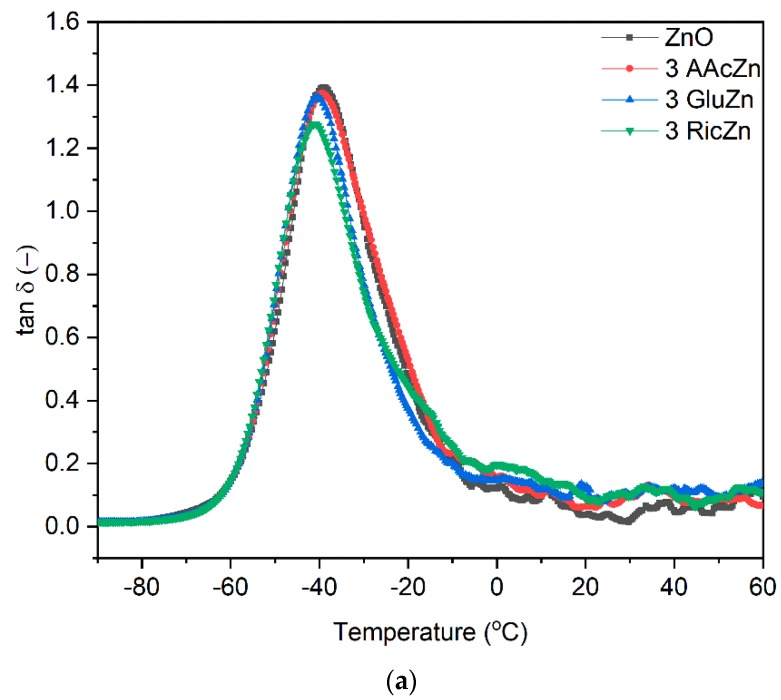

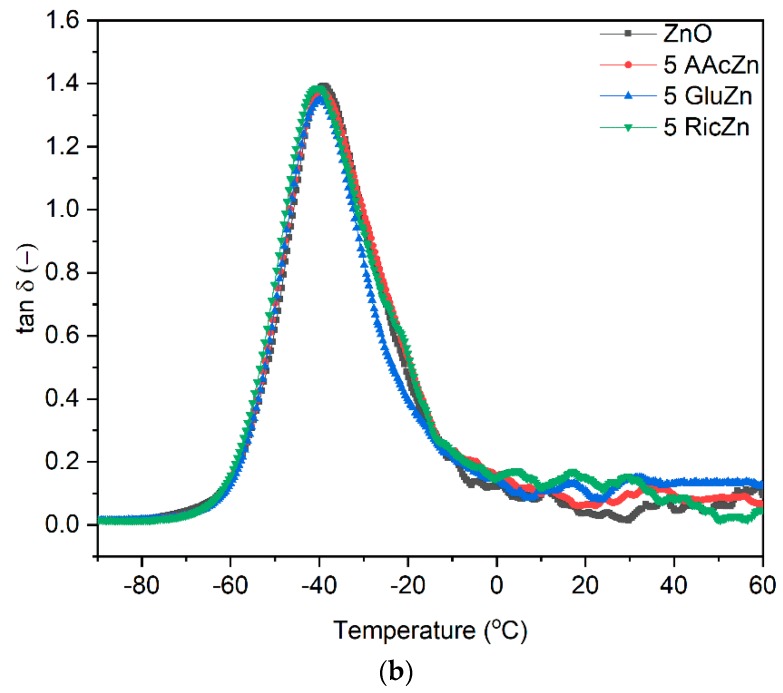
Loss factor (tan δ) versus temperature of SBR vulcanizates with varying activators: (**a**) 3 phr, (**b**) 5 phr.

Dynamic mechanical properties play an essential role in technological applications of SBR compounds due to the fact that rubber products often work under conditions of variable stress and strain. However, regardless of the working conditions, their mechanical properties should be stable during use. Moreover, the ability of rubber composites to dampen vibrations is very important, taking into account their application for manufacturing shock absorbers or elements of vibration dampeners. Therefore, dynamic mechanical analysis (DMA) was employed to determine the influence of organic zinc salts or complexes on the viscoelastic properties of the SBR elastomer and its mechanical loss factor (tan δ), which concerns the ability of the material to dampen vibration. DMA measurements were conducted as a function of temperature to study the influence of organic zinc activators on the glass transition (T_g_) of SBR and its properties in the glassy state and in the rubbery elastic region. The results are presented in Table 6 and Figure 5 and Figure 6.

The maximum tan δ in the DMA curve corresponds to the *T*_g_ temperature of SBR, which was approximately –38.8 °C for the vulcanizate containing ZnO. Organic zinc activators did not significantly affect the temperature at which the elastomer went from a hard, glassy state to a rubbery state. It is commonly known that *T*_g_ depends on the crosslink density of vulcanizates and increases with crosslinks density due to less molecular mobility of the elastomer chains between the crosslinks. The type and content of organic activator had no significant influence on the crosslink density of vulcanizates and thus on the *T*_g_ of SBR. The values of *T*_g_ obtained for the reference vulcanizate with ZnO and for elastomers containing organic zinc salts or complexes were within the range of measurement error.

Regarding the loss factor tan δ, organic zinc activators, especially RicZn, slightly reduced the value of tan δ at *T*_g_, but the opposite effect on tan δ was achieved in the rubbery elastic region. The values of tan δ for SBR containing organic zinc activators were in the temperature range of 25–50 °C even 2–3 times as high as for elastomers conventionally crosslinked with microsized ZnO. This resulted from lower values of the storage modulus E’ of SBR containing alternative activators (Figure 5), which depends on the crosslink density of vulcanizates and decreases proportionally with the number of crosslinks in the elastomer network [38]. Therefore, organic zinc salts or complexes could be expected to improve the damping properties of SBR in the rubbery state. This is another advantage of the use of such vulcanization activators in addition to improved tensile strength and elasticity of vulcanizates. Moreover, vulcanizates containing organic zinc activators demonstrated stable dynamic properties in the temperature range of 25–60 °C, since the loss factor tan δ did not change considerably as the temperature increased.

### 3.4. Dispersion of Curatives and Filler in the SBR Elastomer

**Figure 7:**
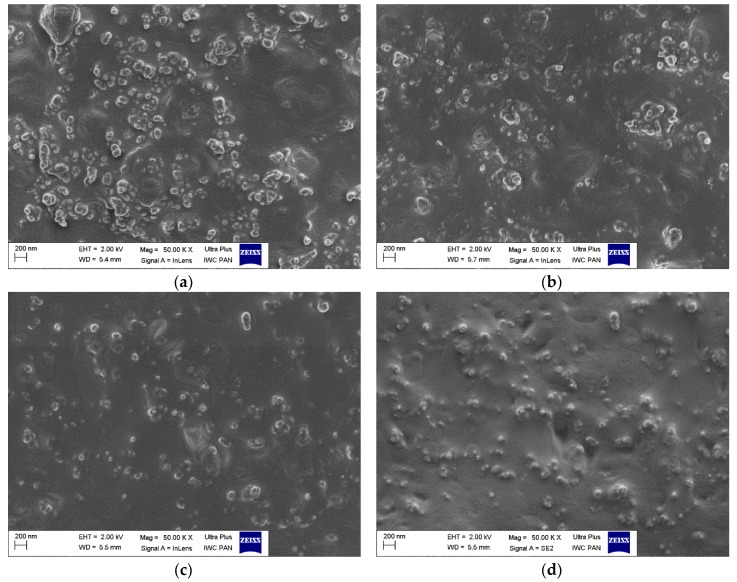
Scanning electron microscopy (SEM) images of SBR vulcanizates with 5 phr of varying activators: (**a**) ZnO, (**b**) AAcZn, (**c**) GluZn, (**d**) RicZn.

According to the mechanism of sulfur vulcanization proposed by Heideman [1] or Nieuwenhuizen [39], the surface of the activator acts as a catalytic reaction template by activating and joining reactants. Accelerator particles, sulfur, and fatty acids diffuse through the elastomer matrix and are adsorbed onto the activator surface, forming intermediate reactive complexes. Therefore, the contact between particles of activators and accelerators in the elastomer matrix should be maximized to enhance the efficiency of the activator during vulcanization. This contact depends on the activator’s particle shape or size. However, the dispersion of activator particles in an elastomer is also very important for the activation of sulfur vulcanization. The agglomeration of activator particles reduces their contact surface with other components of the crosslinking system, and consequently the efficiency of vulcanization. Agglomeration of filler particles reduces its reinforcing effect, since agglomerates may be responsible for local concentration of stresses in vulcanizates subjected to external deformations. Therefore, SEM images were taken to estimate the dispersion degree of curatives and filler particles in the SBR elastomer. Results are presented in Figure 7a–d. 

By analyzing the SEM image of the reference vulcanizate containing ZnO, it was seen that the curatives and filler particles were not homogeneously dispersed in the SBR elastomer. Agglomerates several micrometers in size consisting of nanosized primary particles were created (Figure 7a). However, the agglomerates exhibited good adhesion to the elastomer being surrounded by an elastomeric film that penetrated between the particles, resulting in the good wettability of the agglomerates by elastomer matrix. SEM images showed more uniform dispersion of the curatives and filler particles in the SBR elastomer containing organic zinc activators, compared to vulcanizate with ZnO. In the case of the AAcZn-containing vulcanizate, small aggregates of particles with a size below 1 µm were quite homogeneously dispersed and well wetted by the elastomer matrix (Figure 7b). Regarding the GluZn-containing vulcanizate, the dispersion of particles in the SBR was even better than for vulcanizate with AAcZn, and no agglomeration was observed (Figure 7c). It should be noticed that the most homogeneous dispersion of the curatives and filler and the best adhesion of dispersed particles to the elastomer was obtained for the RicZn-containing vulcanizate. In the SEM image presented in Figure 7d, only individual particles of approximately 200 nm embedded in the elastomer matrix could be seen. The results of SEM analysis show that not only the change in crosslinks density but also the improvement in the dispersion of the curatives and filler particles could have contributed to the increase in tensile strength, compared to the reference vulcanizate containing ZnO.

### 3.5. Thermal Stability of SBR Vulcanizates

**Figure 8:**
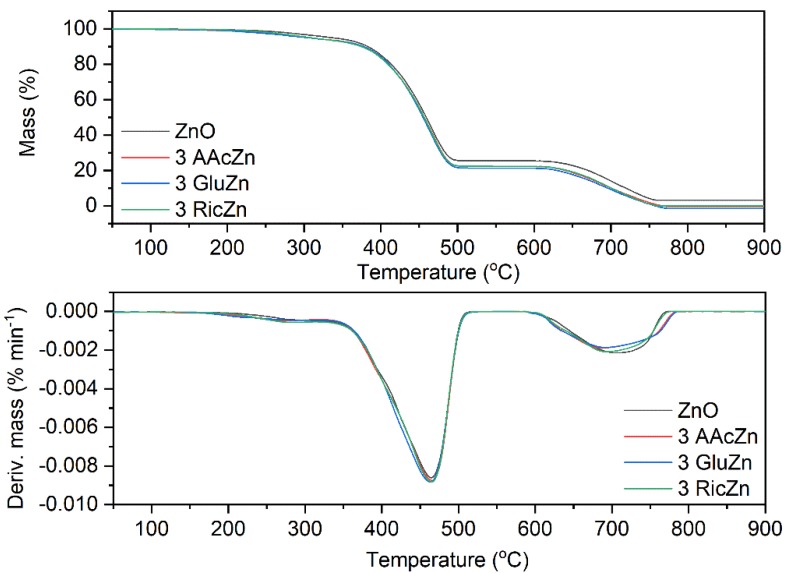
Thermogravimetric (TG) and differential thermogravimetric (DTG) curves of SBR vulcanizates with 3 phr of varying activators.

**Figure 9:**
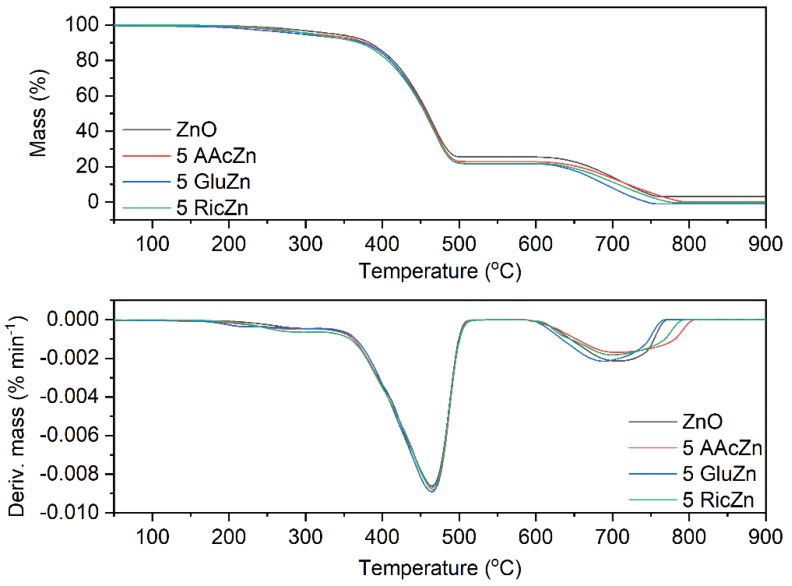
TG and DTG curves of SBR vulcanizates with 5 phr of varying activators.

TG analysis was performed in order to characterize the influence of organic zinc activators and reduction of the zinc ion content in the SBR compounds on the thermal stability of vulcanizates. The temperature required for 5% mass loss during decomposition (*T*_5%_) was determined and taken as the onset decomposition temperature. Furthermore, the temperature of the peak of the differential thermogravimetric (DTG) curves (T_DTG_) was studied. The results are presented in Figure 8 and Figure 9 and summarized in Table 7.

The TG measurements of vulcanizates were performed in two stages. First, samples were heated from 25 to 600 °C in an argon atmosphere to achieve a mass loss corresponding to pyrolysis of the elastomer and thermal decomposition of organic additives, such as vulcanization accelerators or activators in the case of vulcanizates containing organic zinc salts or complexes. Therefore, the mass loss in this temperature range was approximately 2%–3% higher for SBR crosslinked with organic zinc activators compared with the reference vulcanizate with microsized ZnO. At a temperature above 600 °C, the measurement atmosphere was changed into air, so the mass losses in the temperature range of 600–900 °C resulted from the burning of carbon black, which was used as a filler of rubber compounds and was similar for all vulcanizates. At 900 °C, the residue consisted of ash and ZnO in the case of the reference vulcanizate. 

Upon analyzing the thermal stability, the onset decomposition temperature *T*_5%_ determined for vulcanizate containing ZnO was 325 °C. SBR crosslinked with organic zinc activators exhibited approximately 20 °C lower *T*_5%_ temperatures. On the other hand, organic zinc salts or complexes did not influence the T_DTG_ values, so the temperature of elastomer pyrolysis, which led to major decomposition of the vulcanizates. Therefore, it was concluded that the decrease in *T*_5%_ temperature of vulcanizates was due to lower stability of organic zinc activators, the decomposition of which preceded the pyrolysis of the elastomer. To confirm this conclusion, the onset decomposition temperature *T*_5%_ of organic zinc activators was determined (Table 7). The lowest thermal stability was exhibited by the GluZn, which started decomposition at temperatures above 100 °C. The *T*_5%_ temperature of the most thermally stable organic activator RicZn was 194 °C, while the total weight loss of zinc oxide in the studied temperature range was about 1% and was determined at a temperature approximately 300 °C, so at the temperature similar to *T*_5%_ of the vulcanizates. A slight reduction in the *T*_5%_ temperature resulting from the use of organic zinc activators should not affect the potential technological applications of tested materials, since they are thermally stable up to a temperature of 300 °C.

### 3.6. Resistance of SBR Vulcanizates to Thermo-Oxidative Aging

**Figure 10:**
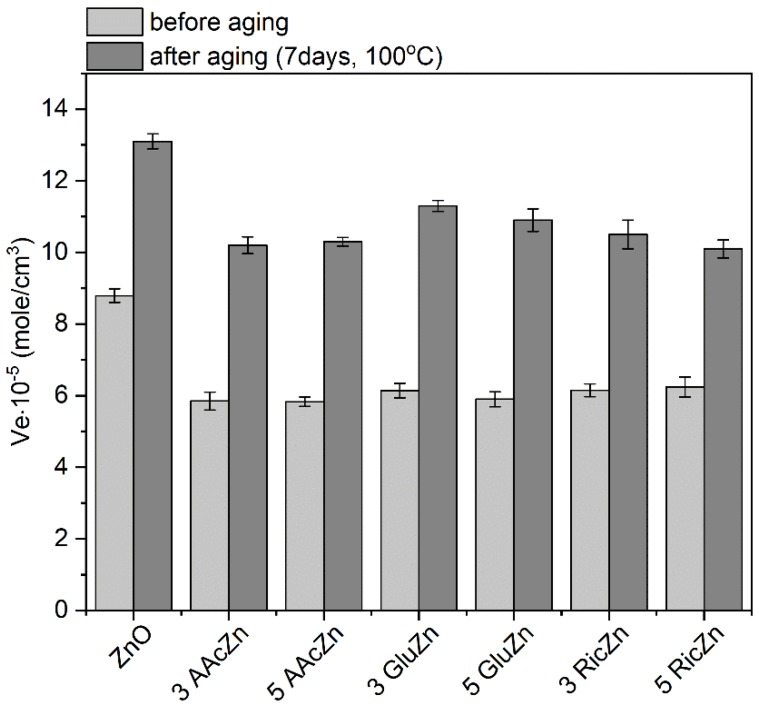
Effect of thermo-oxidative aging on the crosslink density of SBR vulcanizates with varying activators.

**Figure 11:**
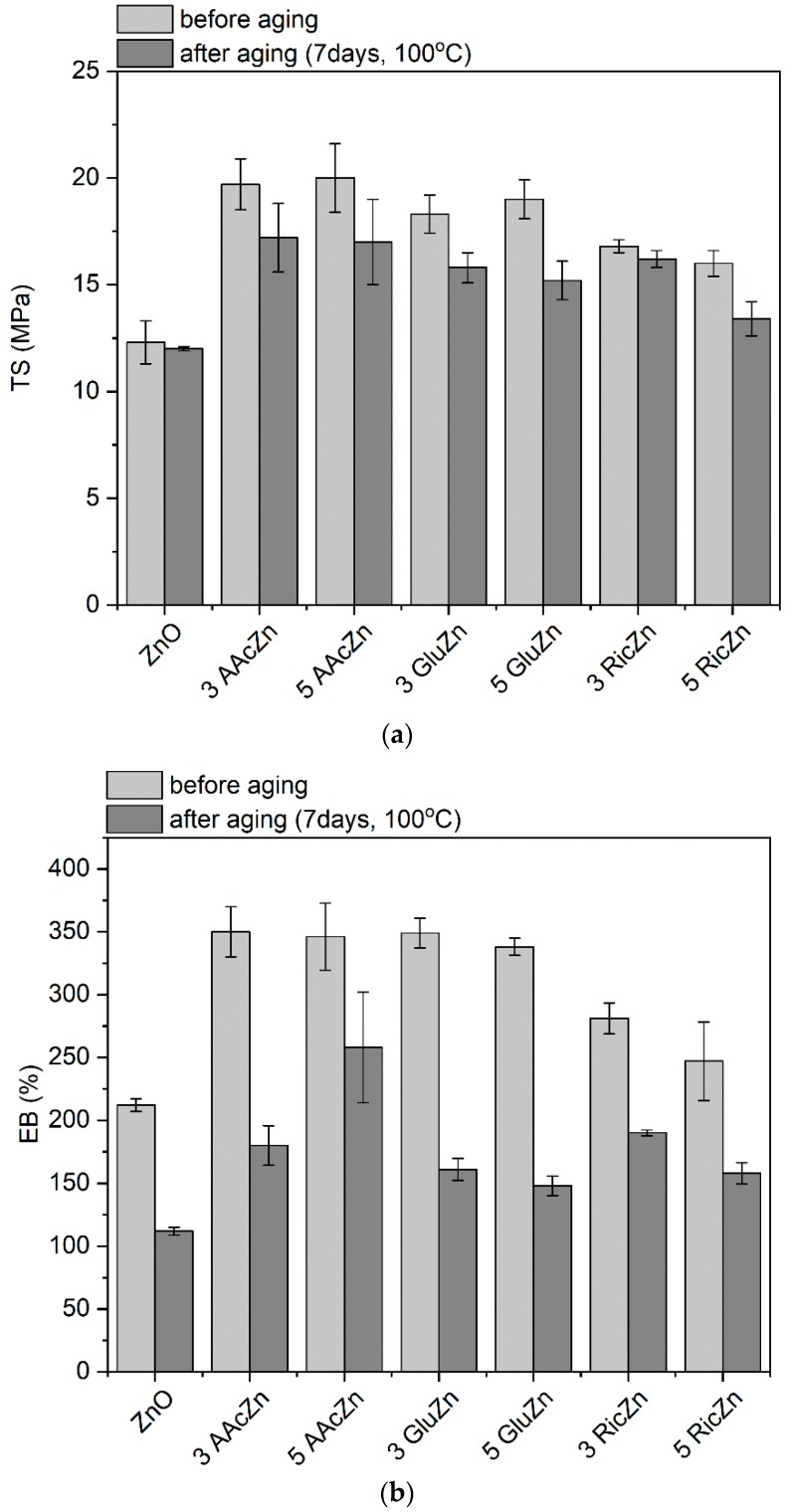
Effect of thermo-oxidative aging on the mechanical properties of SBR vulcanizates with varying activators: (**a**) tensile strenght (**b**) elongation at break.

Having established that the application of organic zinc activators alternatively to microsized ZnO did not deteriorate the mechanical properties of vulcanizates under static and dynamic conditions, we then examined their effects on the resistance of the SBR composites to thermo-oxidative aging. Vulcanizates were exposed to the conditions of thermo-oxidative aging at 100 °C for seven days. Next, their crosslink densities and tensile properties were studied and compared with the values obtained before the aging process. The results are presented in Figure 10 and Figure 11.

Under the influence of an elevated temperature, further crosslinking of the elastomer occurred, resulting in an increased crosslink density of the vulcanizates (Figure 10). A similar effect of thermooxidative aging on the crosslink density has been reported for other elastomers [31,40,41]. It should be noted that SBR compounds were vulcanized using optimal vulcanization time t_95_, so the time required for the torque to reach 95% of the maximum achievable torque. This value relates to the time necessary for the cured rubber to achieve optimal properties. However, this does not mean that the curatives have been totally consumed [42]. Reactive species or radicals after the vulcanization process may still be present in the elastomer and initiate further crosslinking under prolonged exposure to high temperatures. Similar changes in the crosslink density as a result of aging were achieved for the reference vulcanizate containing microsized ZnO and for elastomer with organic zinc activators. The process of thermo-oxidative aging did not considerably affect the tensile strength of elastomers crosslinked with ZnO, whereas the elongation at break was reduced by 100% owing to the significant increase in the crosslink density (Figure 11). A similar effect of elevated temperature on TS and EB was observed for the RicZn containing vulcanizate. On the other hand, the tensile strength after aging of the vulcanizates containing other organic zinc activators decreased by 3–4 MPa compared to the TS values before thermo-oxidative aging. The values of their elongation at break were approximately 160%–190% lower than for vulcanizates before the aging process.

Based on the collected data, the aging factor (AF) was calculated to quantitatively estimate the changes in the mechanical properties of the vulcanizates due to thermo-oxidative aging (Table 8). This factor is a measurement of the changes in the deformation energy of a sample caused by the aging process. The smaller the changes in the mechanical properties of the vulcanizates due to the aging process, the higher the AF, and as a result, the better resistance of the elastomer to aging.

SBR vulcanizates are susceptible to degradation under long-term exposure to elevated temperature. The AF for the reference vulcanizate with ZnO was 0.52. By analyzing the effect of organic zinc activators on the aging factor, the highest AF values were found to occur for the vulcanizates containing RicZn—0.65 and 0.60, respectively. The application of GluZn activator slightly reduced the aging factor to approximately 0.4. Regarding the measurement error, the AF values for AAcZn-containing vulcanizates were similar to those of the reference sample with microsized ZnO. Taking into account the range of changes in the aging factor of vulcanizates containing organic zinc activators in comparison with a conventionally crosslinked elastomer, it can be concluded that organic activators did not considerably affect the resistance of SBR to thermo-oxidative aging.

## 4. Conclusions

Zinc acetylacetonate and organic zinc salts, such as zinc gluconate and zinc ricinoleate, were used to activate the sulfur vulcanization of SBR.

Organic zinc activators are good alternatives to zinc oxide for the vulcanization of SBR without any detrimental effect on the vulcanization time or temperature. Owing to the 70–90 wt % smaller amount of zinc ions introduced into the rubber compared with the conventional microsized ZnO, vulcanizates containing organic zinc activators exhibit lower crosslink densities. However, the decrease in the crosslink density of vulcanizates does not deteriorate their tensile properties. Vulcanizates containing organic zinc activators show higher tensile strength and elasticity compared to the SBR vulcanizate with microsized ZnO. Additionally, these vulcanizates showed better damping properties in the rubbery elastic region than conventionally crosslinked elastomers. This is another advantage of the application of the proposed alternative activators. On the other hand, vulcanizates obtained with organic zinc activators were softer and less stiff, and thus demonstrated slightly different mechanical behavior than the rubber cured with ZnO. Therefore, these vulcanizates cannot be regarded as a replacement for ZnO-cured material in certain applications.

Organic zinc salts or complexes do not considerably influence the resistance of SBR to thermo-oxidative aging. A slight reduction of thermal stability resulting from their use should not affect the potential technological applications of tested elastomer materials.

The content of organic zinc activators has no significant effect on the time and temperature of SBR vulcanization, on the crosslink density and properties of vulcanizates; therefore, their amount in rubber compounds could be reduced to 3 phr. Consequently, the amount of zinc ions applied is reduced by 70–90 wt % compared to zinc oxide. This is particularly relevant, as zinc released to the environment is toxic to aquatic organisms and may cause long-term detrimental effects in the aquatic environment. It should be noted that the rubber industry has a huge global impact. The use of rubber products in the world is constantly growing, from tires and automotive accessories to industrial rubber products, construction applications, medical products, and footwear. Therefore, reducing the use of Zn^2+^ ions in rubber products is an important ecological goal. The importance of this achievement is underlined by the directives of the European Union, which require the amount of zinc in rubber products to be reduced.

## Figures and Tables

**Table 1:** General composition of styrene–butadiene elastomer (SBR) compounds, parts per hundred of rubber (phr) (*alternatively AAcZn, GluZn, RicZn). CBS: N-cyclohexyl-2-benzothiazolesulfenamide; MBT: 2-mercaptobenzothiazole.

Components	Reference Sample	Rubber Compounds with Organic Zinc Activators
SBR	100	100
MBT	1	1
CBS	1	1
Sulfur	2	2
Carbon black	30	30
ZnO	5	-
Organic zinc activator*	-	3; 5

**Table 2:** Cure characteristics of SBR compounds at 160 °C and crosslink densities of vulcanizates (ΔS—torque increment, t_05_—scorch time, t_95_—optimal vulcanization time, ν_e_—crosslink density, standard deviations: ΔS ±1.5 dNm, t_05_ ±0.4 min., t_95_ ±0.5 min., ν_e_ ±0.2 × 10^–5^ mole/cm^3^).

Activator	Amount of Activator (phr)	ΔS (dNm)	t_05_ (min)	t_95_ (min)	ν_e_∙10^−5^ (mole/cm^3^)	Zn^2+^ Amount in SBR Compound (mmole/100 g SBR)
ZnO	5	14.3	1.1	3.0	8.8	61.5
AAcZn	3	8.5	0.9	3.0	5.9	11.4
	5	7.9	1.0	3.0	5.8	19.0
GluZn	3	8.7	1.6	4.0	6.1	6.6
	5	8.3	2.0	4.0	5.9	11.0
RicZn	3	9.2	1.2	2.5	6.2	4.5
	5	9.5	1.6	3.0	6.3	7.6

**Table 3:** Temperature and enthalpy (ΔH) of SBR vulcanization measured by DSC (standard deviations: temperature ±2 °C; ΔH ± 1.7 J/g).

Activator	Amount of Activator (phr)	Temperature of Vulcanization (°C)	Temperature of Peak (°C)	∆H (J/g)
ZnO	5	159–218	171	10.6
AAcZn	3	158–229	178	10.3
	5	155–224	184	9.9
GluZn	3	154–232	175	11.9
	5	159–232	184	14.7
RicZn	3	153–220	167	12.0
	5	153–223	171	12.6

**Table 4:** Mechanical properties and hardness of SBR vulcanizates (SE_100_—modulus at a relative elongation of 100%, TS—tensile strength, EB—elongation at break, H—hardness, standard deviations: SE_100_ ±0.2 MPa, TS ±0.9 MPa, EB ±15%, H ±2 ShA).

Activator	Amount of Activator (phr)	SE_100_ (MPa)	TS (MPa)	EB (%)	H (ShA)
ZnO	5	4.1	12.3	212	55
AAcZn	3	2.3	19.7	350	46
	5	2.5	20.0	346	47
GluZn	3	2.7	18.3	349	48
	5	2.6	19.0	338	49
RicZn	3	2.9	16.8	281	45
	5	2.8	16.0	247	44

**Table 5:** Permanent set of SBR vulcanizates (standard deviation ±2%).

Activator	Amount of Activator (phr)	Permanent Set (%)
ZnO	5	22
AAcZn	3	29
	5	27
GluZn	3	37
	5	35
RicZn	3	30
	5	32

**Table 6:** Glass transition temperature (*T*_g_) and loss factor (tan δ) of SBR vulcanizates (standard deviations: *T*_g_ ±0.5 °C, tan δ ±0.06).

Activator	Amount of Activator (phr)	*T*_g_ (°C)	tan δ at *T*_g_ (-)	tan δ at 25 °C (-)	tan δ at 50 °C (-)
ZnO	5	−38.8	1.39	0.04	0.03
AAcZn	3	−39.2	1.38	0.11	0.09
	5	−39.4	1.37	0.09	0.08
GluZn	3	−39.6	1.36	0.13	0.10
	5	−39.8	1.35	0.11	0.12
RicZn	3	−39.9	1.30	0.11	0.10
	5	−40.5	1.36	0.13	0.06

**Table 7:** Decomposition temperature at 5% (*T*_5%_) mass change, differential thermogravimetric (DTG) peak temperature (*T*_DTG_), and total mass loss (Δm) during thermal decomposition of SBR vulcanizates (standard deviations: *T*_5%_ ±1.7 °C, *T*_DTG_ ±1.1 °C, Δm ±1.5%).

Activator	*T*_5%_ of Activator (°C)	Amount of Activator (phr)	*T*_5%_ (°C)	*T*_DTG_ (°C)	∆m (25–600 °C) (%)	∆m (600–900 °C) (%)	Residue at 900 °C (%)
ZnO	-	5	325	469	74.4	22.3	3.3
AAcZn	140	3	303	465	76.4	22.5	1.1
		5	309	467	77.2	22.7	0.1
GluZn	106	3	303	465	76.3	22.6	1.1
		5	301	467	77.3	22.5	0.5
RicZn	194	3	307	467	76.6	22.3	1.1
		5	305	467	77.4	22.2	0.4

**Table 8:** Aging factor (AF) of SBR vulcanizates (standard deviation: AF ±0.07).

Activator	Amount of Activator (phr)	AF (-)
ZnO	5	0.52
AAcZn	3	0.45
	5	0.46
GluZn	3	0.40
	5	0.36
RicZn	3	0.65
	5	0.60
